# Prediction of Indoor Air Exposure from Outdoor Air Quality Using an Artificial Neural Network Model for Inner City Commercial Buildings

**DOI:** 10.3390/ijerph121214975

**Published:** 2015-12-01

**Authors:** Avril Challoner, Francesco Pilla, Laurence Gill

**Affiliations:** Department of Civil, Structural and Environmental Engineering, Trinity College Dublin, Dublin 2, Ireland; avrilchalloner@gmail.com (A.C.); fpilla@tcd.ie (F.P.)

**Keywords:** indoor/outdoor air quality, Geographical Information System (GIS) modelling, data mining, artificial neural networks, pollution, health impacts

## Abstract

NO_2_ and particulate matter are the air pollutants of most concern in Ireland, with possible links to the higher respiratory and cardiovascular mortality and morbidity rates found in the country compared to the rest of Europe. Currently, air quality limits in Europe only cover outdoor environments yet the quality of indoor air is an essential determinant of a person’s well-being, especially since the average person spends more than 90% of their time indoors. The modelling conducted in this research aims to provide a framework for epidemiological studies by the use of publically available data from fixed outdoor monitoring stations to predict indoor air quality more accurately. Predictions are made using two modelling techniques, the Personal-exposure Activity Location Model (PALM), to predict outdoor air quality at a particular building, and Artificial Neural Networks, to model the indoor/outdoor relationship of the building. This joint approach has been used to predict indoor air concentrations for three inner city commercial buildings in Dublin, where parallel indoor and outdoor diurnal monitoring had been carried out on site. This modelling methodology has been shown to provide reasonable predictions of average NO_2_ indoor air quality compared to the monitored data, but did not perform well in the prediction of indoor PM_2.5_ concentrations. Hence, this approach could be used to determine NO_2_ exposures more rigorously of those who work and/or live in the city centre, which can then be linked to potential health impacts.

## 1. Introduction

The United Nations Urban Environment Unit associates up to one million premature deaths annually to urban air pollution and over 90% of the air pollution in developing cities has been linked with poor quality vehicles [1]. Illnesses to which poor outdoor air quality has been attributed include: cancers of the bladder, kidney, stomach, oral cavity, pharynx and larynx, multiple myeloma, leukaemia, Hodgkin’s disease, and non-Hodgkin's lymphoma [2].

The predictive models developed in this research were based upon measured concentrations of PM_2.5_ and NO_2_ inside and outside commercial buildings in Dublin, Ireland [3]. A study into air pollution in 26 cities across Europe [4] noted that Dublin, with a population of approximately 1.2 million [5] in an area of 290 km^2^, has comparatively low concentrations of air pollutants, such as NO_2_ and PM_2.5_, which were within EU limits. However, a recent report by the Irish Environmental Protection Authority (EPA) stated that NO_2_ and particulate matter were the two pollutants of most concern in Ireland [6] which may be due to the high respiratory and cardiovascular mortality and morbidity rates in Ireland compared to most of the rest of Europe [1,7]. Although these illnesses may not be directly caused by poor air quality, they may be worsened by it. In particular, respiratory illness (such as asthma and bronchitis) is the third most reported illness in Ireland after cardiovascular and musculoskeletal diseases. Sufferers of respiratory illnesses are a high-risk group with respect to air quality and are adversely impacted with declining air quality faster than the general population. For example, the loss of working hours due to asthma has been estimated at three days per adult and is estimated to cost the Irish economy €16.6 million [8]. Statistically significant increases in hospital admissions have been recorded with increased periods of NO_2_ in Athens [9], which concur with a calculated 0.5% increase expected for every 10 μg·m^−3^ increase in NO_2_ concentrations [10]. Oxides of Nitrogen (NO_x_) and PM_2.5_ put strain on the cardiovascular and respiratory systems, thereby aggravating illness, and so any reduction in concentrations, regardless of limit values, should benefit a population with high rates of such illnesses.

Currently, air quality limits in Europe only cover outdoor environments, yet the quality of indoor air is an essential determinant a person’s well-being, especially since the average person spends more than 90% of their time indoors [11,12]. Indoor health was not considered when comparing European PM_2.5_ and NO_2_ legislative concentrations, yet poor indoor air quality has been associated with symptoms like headaches, fatigue, trouble concentrating, and irritation of the eyes, nose, throat and lungs, all of which effect the productivity of a workforce [2,13,14,15]. Most cities now have a number of ambient air quality monitoring stations but studies have found that such ambient outdoor measurements can prove to be a poor predictor of personal work-day exposure, with the higher personal exposures often due to increased indoor concentrations of the measured pollutant. For example the EXPOLIS study found median correlations of personal exposure and outdoor monitoring of PM_2.5_ ranging from 0.39 to 0.91 across Europe [16,17]. The link between indoor and outdoor air quality in commercial buildings was also studied by Mosqueron *et al.* [18], who found a correlation of *r* = 0.05 when comparing urban background concentrations with in-office concentrations for NO_2_ and PM_2.5_ in Paris. Zeger *et al.* [19] also previously found that fixed site monitoring was not ideal for calculating exposure. A European wide study known as AIRMEX reported that indoor concentrations of Volatile Organic Compounds (VOCs) and PM_10_ in two Dublin city centre offices in May 2007 were often higher than outdoor concentrations [20].

There has been much recent research into the use of different modelling approaches to predict a variety of different outdoor air pollutant concentrations at higher resolutions for specific locations in the urban environment to improve upon the relatively sparse ambient monitoring data that is normally available, see for example [21,22,23,24,25]. However, there have been much fewer studies that have tried to predict indoor air quality from the local outdoor conditions in such an urban environment. Hence, this research aims to provide a methodology based upon modelling which can use publically available data from fixed site monitoring stations in order to predict indoor air quality more accurately. Predictions are made using two modelling techniques. Initially, Artificial Neural Networks (ANN) models were developed to determine the dynamic relationship between the measured outdoor and indoor air quality of several monitored buildings. The Personal-exposure Activity Location Model (PALM) model [26,27] was then used to predict the outdoor air quality at any particular building in the city and thus provide an input into the ANN models to predict indoor air quality. This approach ultimately provides predicted indoor air concentrations, which can then be used to determine urban workers’ pollutant exposures more rigorously. This data could then be linked to future epidemiological studies, for example the incidence of respiratory illnesses of those who work and/or live in the city centre.

## 2. Experimental Section

### 2.1. Experimental Data

As part of a wider research project, summarized in Challoner and Gill [3], ten commercial buildings were chosen for air quality monitoring, all located along busy street canyons in Dublin’s city centre. Three of these buildings were chosen for this more detailed study which has developed ANN based models to predict indoor air quality from of outdoor air quality measurements, as discussed later. These buildings were chose due to their proximity to each other, on one side of a heavily trafficked inner city street (Pearse Street), in addition to having different ventilation and use attributes: two are mechanically ventilated and the third is naturally ventilated (see Table 1 for details). The indoor monitoring at the first of the mechanically ventilated buildings (M_c_2) took place in a small office space (2.9 m × 4.2 m plan and 4.5 m high) while the indoor monitoring at the second mechanically ventilated building (M_c_3) was in a large open gallery space (room volume 702 m^3^). The ventilation systems for both buildings were controlled upon a set-point temperature and humidity matrix rather than on a specific number of air changes per hour. The naturally ventilated space (N_t_2) was a medium sized office (9.7 m × 4.0 m plan and 4.0 m high) with six occupants. PM_2.5_ and NO_2_ concentrations were measured simultaneously indoors and outdoors of the different buildings (shops and offices). Outdoor concentrations were measured in two locations either at ground level outside the building or at the air intake of the building’s ventilation system. For example, for the first monitoring period (Run 1) at M_c_2, outdoor air quality was monitored at roof level whilst for Run 2 outdoor air quality was monitored at ground level (as detailed in Table 1). For Run 2 at M_c_3 an extra set of monitors was resourced to enable outdoor monitoring to be conducted at roof and ground level simultaneously to the indoor monitoring. Both indoor and outdoor measurements were taken at a height of 1 to 1.5 m above ground level.

**Table 1:** Monitoring sites summary and details.

Site No.	Building Type	Vent. Type	Age (Years)	Opening (h)	Run 1		Run 2	
N_t_2	Office	Nat.	~120	10 a.m.–6 p.m.	26–29 April 2011	Ground	27 June–1 July 2011	Ground
M_c_2	Office	Mech.	~5	8 a.m.–6 p.m.	6–9 July 2010	Roof	12–15 July 2010	Ground
M_c_3	Shop	Mech.	~5	8 a.m.–8 p.m.	13–16 December 2010	Ground	27–31 March 2011	Roof/Ground

The indoor and outdoor measurements of PM_2.5_ were measured by two identical Haz-Dust monitors (Environmental Devices Corporation, EPAM-5000, Haz-Dust) set at a flow rate of 2 L·min^−1^. For NOx, two Teledyne, M200 monitors (which work on the principle of chemiluminescence) were used to measure NO and NO_2_: a M200E model was used for outdoor monitoring with a limit of detection of less than 1 ppb and a M200EU model used for indoor monitoring with limit of detection of 0.05 ppb. Both monitors were set to a flow rate of 0.479 L·min^−1^. Weather data were sourced from the national meteorological (Met Eireann) monitoring stations located in Phoenix Park and Dublin Airport. Full details of the results are contained in Challoner and Gill [3].

### 2.2. Artificial Neural Network Model

An artificial neural network (ANN) is a robust non-linear computational method which was originally designed to emulate biological nervous systems but has since been applied to many fields of study including air pollution [28,29]. ANNs do not have pre-defined assumptions such as prior hypotheses regarding variable relations; they have a low sensitivity to error term assumptions and a high tolerance to noise. ANN makes use of a complex combination of weights and functions to convert input variables into an output (prediction). It can be employed to examine relationships in complex non-linear data sets in the same way as conventional statistical techniques, but without many of the parametric restrictions about the nature of the data relationships. ANNs use previously collected times series data (e.g., indoor concentrations and outdoor meteorological data in the case of this research), that the model is being developed to predict. In the current study, the Levenberg-Marquardt Algorithm [30,31], a type of feed-forward ANN, is utilised for the modelling procedure Equation (1). This algorithm provides a numerical solution to the problem of minimising a function, generally nonlinear, over a space of parameters of the function. The Levenberg-Marquardt Algorithm (LMA) interpolates between the Gauss-Newton Algorithm [32,33] and the method of gradient descent, which is a first order optimisation algorithm.

(1) $$\left(J^{T}J+\lambda diag\left(J^{T}J\right)\right)\delta =J^{T}\left[y-f\left(\beta \right)\right]$$

where:

*J*—Local gradient of f with respect to β
β—Parameters
*y*—Independent and dependent variables
δ—Increment

The ANN has an inputs layer, at least one neuron layer (although usually a group of interconnecting neurons are present) and an outputs layer [34]. Using input data the ANN is “trained” by inputting a set of “target” values (in this case the indoor air quality concentrations), which the ANN should achieve by processing the input data. Once trained and tested, the ANN can be applied widely in a number of applications because of their fascinating characteristics of robustness, fault tolerance, adaptive learning ability and massive parallel processing capabilities. For example, ANNs have been used for time series prediction of air pollution levels at monitoring station locations [35], at street level [24,36] and at locations of particular interest such as road intersections [25].

#### Input Parameters

A Matlab toolbox called “Neural Network Time series Tool” using a non-linear auto-regression with external input networks (NARX) modelling technique was chosen to calculate interactions between indoor and outdoor concentrations of PM_2.5_ and NO_2_, and meteorological data. The NARX network is a two-layer feed forward time delay neural network (TDNN) which uses a sigmoid transfer function in the hidden layer and a linear transfer function in the output layer. In order to train the system, the feedback loops between the output and input (which are usually closed) were opened. A pre-set time lag of two time steps, between input variables and target reactions was initially selected. The input variables chosen were; time of day, barometer level pressure (hPa), sea level pressure (hPa), temperature (°C), relative humidity (%), wind speed (knots), wind direction (knots), Pasquill atmospheric stability class, global solar radiation (j·cm^−2^) and outdoor pollutant concentrations.

The indoor concentration datasets, or targets, were divided into three subsets in order to train, validate and test the Matlab NARX model. The proportion of this division was chosen to be 75% for training, 10% for validation and 15% for testing of the model as used in other studies [25,36]. The idea of training is to pick up on hidden neurons or interactions between the data, which may be a combination of several variations in meteorological data that vary the relationship between indoor and outdoor concentrations. These neurons increase the prediction ability of the ANN over a simple regression. The validation process was then used to further refine the neural network construction and to minimise over-fitting. Validation checks ensured that increases in the accuracy of the network as a result of training were due to increased accuracy over the data set that was not previously seen. Finally, once the Matlab routine had found the best solution to the training and validation of the network, testing of the remaining 15% of data was performed. Testing was carried out in order to confirm the actual predictive power of the network.

### 2.3. Prediction of Outdoor Levels using PALM Model

The PALM-GIS model [27] was used to predict the outdoor pollution levels at the locations of the test sites. The PALM-GIS model uses custom Python scripts to integrate various air dispersion models (such as the Operational Street Pollution Model [37], the General Finite Line Source Model [38] and Gaussian Dispersion models) with a Geographic Information Systems (ArcGIS) platform; the advantage of this solution is that scripts are used to automate the time-consuming and complex GIS workflows, such as the iteration of the modelling procedure for different modelling tests and weather conditions. ArcGIS also allows the user to create a custom user script tool by coding the workflow and the succession of commands. The custom tool can then be easily called and used by any ArcGIS user. This integration aims to provide the researchers, Local Authorities and others with a tool to calculate the concentration levels of air pollutants and to correlate them with other thematic layers, such as land use and population density, in order to link localized peaks in air pollutants with particular activities. As such, the following outcomes were obtained by using dedicated ArcGIS workflows and tools:

(1) Modelled background concentration levels;
(2) Modelled traffic related concentration levels in urban and sub-urban environments;
(3) Modelled industrial sources related concentration levels;
(4) Modelled domestic sources related concentration levels;

The concentration levels were then combined in ArcGIS in order to obtain total concentration levels at the test locations for the periods during the different monitoring runs.

#### Data for PALM Model

The following datasets were used in the models described in the previous section:

(1) Weather data: weather data at an hourly time step was obtained from Met Eireann for the Dublin Airport synoptic stations (located 8 km from the city centre on the north side of the city) for: wind speed, wind direction, temperature, humidity, dew point, atmospheric pressure, rainfall, solar radiation and atmospheric stability classes.
(2) NO_2_ and PM_2.5_ data: daily average NO_2_ and PM_2.5_ concentration levels were sourced from the monitoring stations in the Great Dublin Area, classified as “Background” stations by the Irish EPA.
(3) Traffic data: the traffic data used for the OSPM (Operational Street Pollution Model) model [37] was obtained from Dublin City Council (DCC). DCC monitors traffic continuously at different traffic intersections (critical junctions) around the city. The time resolution is was generally 15 min aggregate data. For the motorways, Port Tunnel, *etc.*, information is collected by The National Road Authority (NRA) and then stored/archived by DCC.
(4) Building geometry and road network: streets and buildings data for the Great Dublin Area were supplied by Dublin City Council in GIS format; as such the initial main challenge in using OSPM in this project is to import these street and buildings data into the environmental software. The buildings and road network were imported in OSPM using AirGIS [39].

### 2.4. Forward Prediction of Indoor Air Quality using Artificial Neural Networks

The training of open networks as previously discussed is a useful method to check if hidden connections between indoor and outdoor air quality and other meteorological factors can be found, therefore increasing the prediction power over that of a simple regression. While this is useful, the real power in the use of an ANN lies in forward prediction. The forward prediction model used the original ANN run at a specific site to train a network as previously discussed. This network was then closed, meaning that no more target (*i.e*., indoor) data could be provided. Once the network was closed, new inputs for the second run, *i.e.*, the outdoor concentrations and meteorological conditions, were used in conjunction with the previously trained network to predict the new indoor concentrations.

The add-on code required three input files (original inputs, original targets and inputs for forward prediction model). Changes to the input delays or hidden networks were specified at this point if required, in addition to changes in the amount of data used for training, validation and testing of the open network. At this point the network was trained using the first run of data, as was done in the previous sections. Once the original network was trained, the code automatically closed the network, which means that no more target data (*i.e*., indoor concentrations) would be provided. The new input data, as calculated in the previous section, for forward predictions was then fed into the trained network and the model predicted the response of the indoor air quality concentrations due to fluctuations in outdoor air quality and weather data.

## 3. Results

### 3.1. Development of ANNs for Individual Sites

ANNs for all three buildings were computed for both NO_2_ and PM_10_ using the real data from the parallel indoor and outdoor monitoring described in Section 2.1. An illustrative set of figures are shown for the first site and other pertinent examples, whilst all other data has been plotted and provided as Supplementary Information (see Figures S1–S17).

#### 3.1.1. NO_2_ Artificial Neural Network Model Performance

##### M_c_2 (*Office*)

The trained data set for M_c_2 run 1, where the outdoor monitoring was located at roof level produced an *R* value of 0.967 for testing, with an overall R of 0.990 for the testing, validation and training periods, as shown on Figures S1 and S2. M_c_2 run 2 (when outdoor monitors were located at ground level) resulted in only 2 errors above 1 ppb, the highest of which occurs at Time = 52, (*i.e*., 52 h into the data set) as shown in Figure S3. The goodness of fit for testing of the newly trained network was *R* = 0.91, with a perfect fit for the training period data.

##### M_c_3 (Mechanically Ventilated Gallery Space)

**Figure 1:**
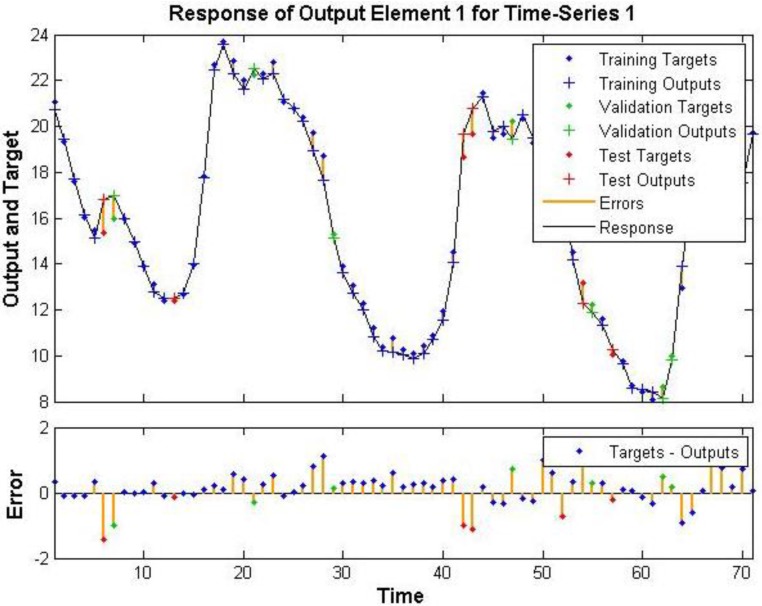
Time series of neural network training M_c_3 run 1 NO_2_.

**Figure 2:**
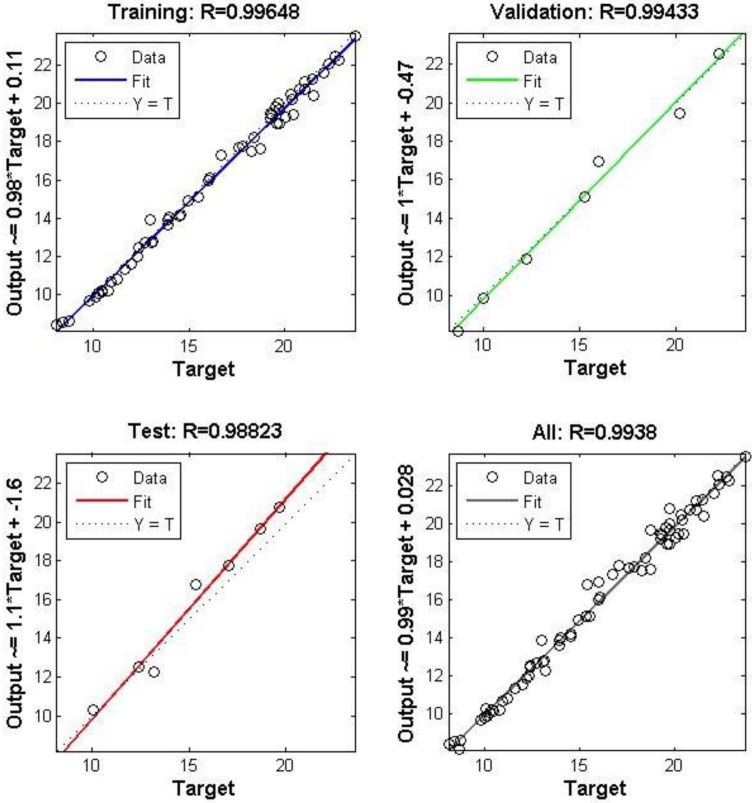
M_c_3 run 1 NO_2_ regression of trained output data set.

The errors for the training, testing and validation phases of M_c_3 run 1 are shown in Figure 1. The neural network has a test data set *R* value of 0.988 (Figure 1 and Figure 2), indicating that a well-trained Neural Network was developed using the meteorological variables and monitored outdoor concentrations of NO_2_ to predict indoor concentrations. Run 2 also produced a very well trained Neural Network with few errors as shown in Figure S4. Figure S5 shows the regression of the training, validation and test data, with test data showing an *R* = 0.964 for M_c_3 run 2.

##### N_t_2 (Naturally Ventilated Office)

The difference between indoor and outdoor concentrations for N_t_2 was significant during both monitoring runs. This was attributed to an unknown process (suspected to be heterogeneous reactions) significantly influencing the data set. In order to ensure this was not due to a once off event, data was collected again several months later for this ground floor naturally ventilated office. For both runs the outdoor data were collected directly outside the main entrance to the office, located less than 10 m from the internal door to the office. Very low indoor concentrations of less than 9 ppb were measured during both runs with outdoor concentrations averaging just under 30 ppb. The training, testing and validation of the ANN for run 1, resulted in a single error above 1 ppb on the second morning near Time = 25 h (see Figure S6). This error occurred as a sharp spike, and similar to Sites M_c_2 and M_c_3 did not influence the trend-line. Errors occurring between Time = 35 h and 50 h however did drag the trend-line down by 1 ppb, which is a considerable error as the range here is only between 3 ppb and 9 ppb. Figures S6 and S7 give an *R* of 0.956 for the testing of the trained neural network.

The errors for run 2, when the monitor was again outside the office door, were less frequent than run 1. The range of indoor NO_2_ data during this run was 0.5 to 4 ppb and, therefore, even errors of 0.5 ppb are significant. In reviewing the individual errors, they occurred at times when sharp spikes in data occurred and have little influence on the trend-line of the data set. Figure S8 shows the regression analysis of the training, validation and test data, with an overall *R* = 0.990 and *R* = 0.81 for the testing phase.

#### 3.1.2. PM_2.5_ Artificial Neural Network Model Performance

In general, the modelling of the PM_2.5_ data showed a higher number of errors, a larger range of errors and lower Pearson’s *R* values for regressions, than the previously described NO_2_ models. The range of hidden neurons was from 10–14 and delays were up to 3 intervals. The delay was set to 30 min.

##### M_c_2 (Mechanically Ventilated Office)

**Figure 3:**
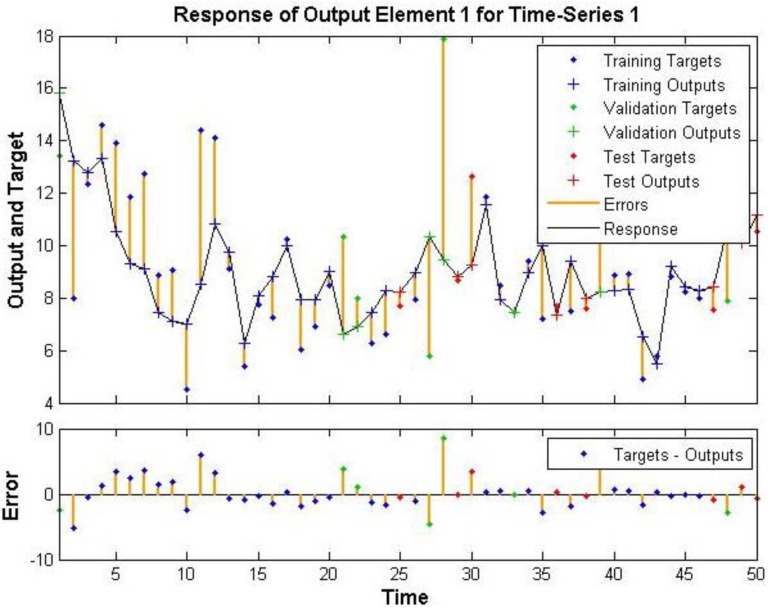
Time series of neural network training M_c_2 run 1 PM_2.5_.

**Figure 4:**
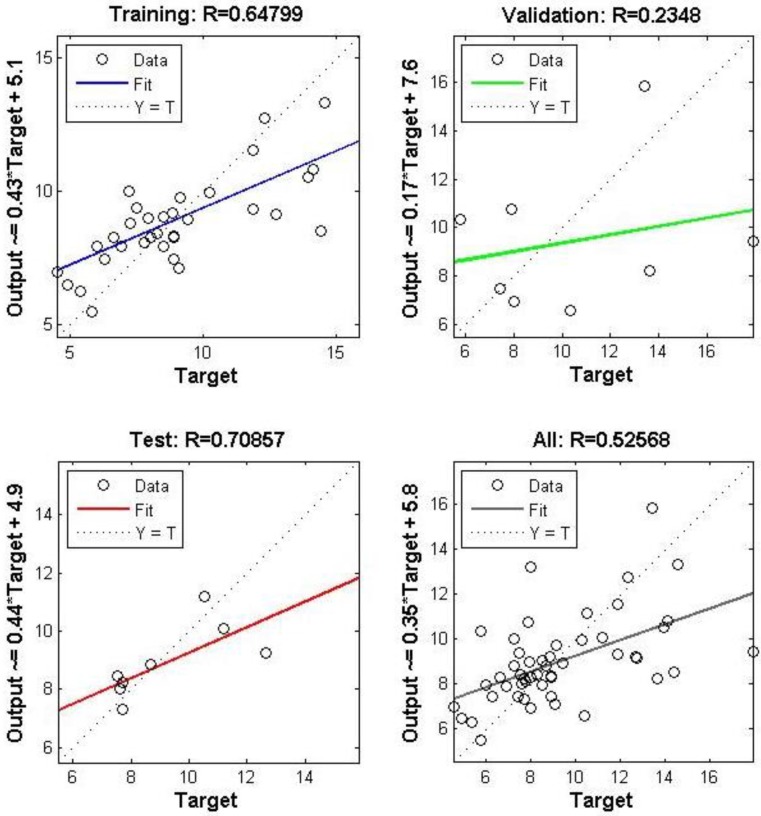
M_c_2 run 1 PM_2.5_ regression of trained output data set.

The ANN model for PM_2.5_ at M_c_2 resulted in some large errors (Figure 3). The monitoring for Run 1 was conducted at the ventilation intake level and the room where indoor monitoring took place had a direct feed to this air intake. Errors for this site range from −8.09 to 4.93 µg·m^−3^. The errors are largest for the validation and training data with only 1 test error point lying outside the range of −1.23 to 0.82 µg·m^−3^. The regression analysis of the neural network also returned poor *R* values compared to the NO_2_ data set for this site of 0.647, 0.234, and 0.708 for training, validation and testing respectively, as shown in Figure 4.

The regression analysis for run 2 yielded better *R* values for training (*R* = 0.984), validation (*R* = 0.780) and testing (*R* = 0.776) than for run 1. These predictions were strong compared to the original regression done between indoor and outdoor air quality concentrations which had an *R*^2^ = 0.11. Errors ranged from −6.86 to 4.62 µg·m^−3^ although all except for six were within the range of −2.02 and 2.20 μg·m^−3^.

##### M_c_3 (Mechanically Ventilated Gallery Space)

Errors for M_c_3 run 1 ranged between −2.73 to 7.37 μg·m^−3^, although only five points were outside the range of −1.14 to 1.52 μg·m^−3^ (Figure S9). The regression analysis on the ANN data in Figure S10 shows *R* values for training, validation and testing of 0.953, −0.011 and 0.864, respectively. The poor validation regression is mainly due to 1 high leverage error; other than this, the validation points produced a relatively good prediction.

During run 2, PM_2.5_ at M_c_3 was monitored simultaneously at roof level, ground level and indoors. This therefore presented an opportunity to see if the extra data *i.e.*, from both ground and roof level simultaneously, improved the ANN performance. The performances of the three different ANNs to predict the indoor data are assessed as follows. Run 2 provided a better *R* value for the regression of the modelled and target values than for run 1, particularly for the validation of the training data (Figure S11). The errors for this run were higher than other runs due to the greater range over which data is spread—between −54.78 and 38.55 μg·m^−3^ (although all instances except for six lie between −25.31 and 4.16 µg·m^−3^).

The roof level data inputs showed a higher *R* value and fewer errors than the street level data, as shown in Figure S12. The errors were within the range of −25.67 to 61.02 μg·m^−3^ but all except five were in the range of −16.54 to 6.27 µg·m^−3^. Most of the larger errors again occur during the peaks, but the roof level data seems to account for a greater number of these than the street level data.

M_c_3 run 2 produced a strong ANN from training using the target data but with high errors due to the significant spike that was seen for the first day and a half of monitoring (see Figure S13). These errors range from −30.05 to 30.95 µg·m^−3^ but all, except seven, lie within the range of −14 to 8.47 µg·m^−3^. An extra input was included in this run as both ventilation intake, or roof level PM_2.5_ data, and ground level data were included, unlike the two previous runs at this site. The inclusion of both roof and ground level data significantly reduced errors during the first day and a half of monitoring during which period the large increase in indoor concentrations were monitored. The large spike at Time = 7 h and magnitude −30.05 occurs for testing data, this error creates a dip in the data between the previous and proceeding data points. Regression analysis for the training, validation and testing of the ANN *versus* the target data yielded high *R* values of 0.992, 0.973, and 0.957, respectively, the high *R* value for testing being due to a high leverage point. These points occurred due to testing and validation points being checked during the first two days, a time when unusually high peaks occurred. The *R* value seems reasonable if these high leverage points were removed.

**Figure 5:**
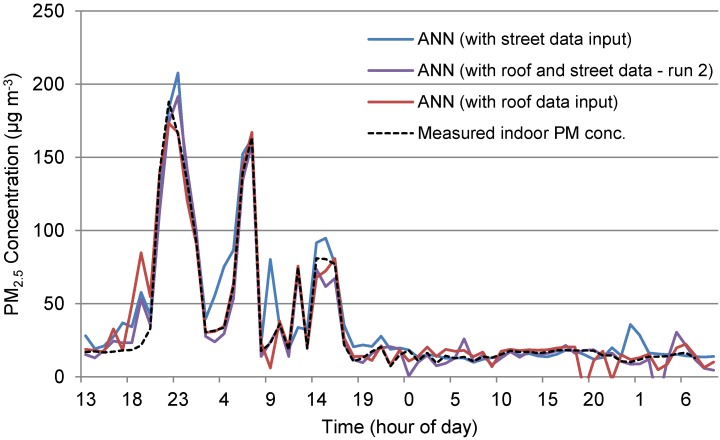
Time series of ANN trained *versus* measured indoor concentrations at M_c_3 (run 2).

The actual indoor data, or target, and the three neural networks trained using data containing roof level data, street level data and a combination of the two as well as meteorological data for each network have been plotted on Figure 5. A comparison of the three trained networks reveals its strong prediction ability with *R* values above 0.95. The results of two sample *t*-tests show estimates of the difference of 1.29, −5.51 and −1.04 between target and roof level, street level and a combination of the two respectively. The two sample *t*-tests found that all three 95% confidence intervals contained zero, therefore, the predicted data using the trained networks for all three situations predicts outputs that have a mean value statistically indifferent from zero. Furthermore, the *R* values found that the target was best predicted by a combination of ground level data and roof level data *R* = 0.976, a lower *R* = 0.965 was found for roof level and finally the lowest R was found between street level and target data. While the combination of roof and ground level combined with meteorological data found the best prediction ability, both street level and roof level found good prediction ability individually.

##### N_t_2 (Naturally Ventilated Office)

N_t_2 run 1 shows a reasonable output for the error during the time series (Figure S14). Errors were between –6.85 and 4.62 μg·m^−3^ although all except five of these error points lie between −2.02 and 2.20 μg·m^−3^. The regression analysis carried out on the 15% of target data set aside for testing yielded an *R* = 0.984, with the validation data yielding a lower *R* = 0.630 and the testing data an *R* = 0.660.

Run 2 for N_t_2 had a very noisy time series, as reflected with a larger number of errors due to the high number of fluctuations (Figure S15). The errors range from −7.21 to 5.03 µg·m^−3^ and have a Gaussian distribution. Regression analysis returned *R* = 1 for training and *R* = 0.936 for testing of the trained data. A stronger *R* of 0.813 was also found for validation compared to run 1.

#### 3.1.3. Discussion of Trained ANNs

The predictions of indoor air quality using the ANNs were much stronger for NO_2_ than PM_2.5_ due to the less erratic NO_2_ time series. The measured NO_2_ time series had more regular diurnal patterns due to the fact that the pollutant is more affected by meteorological variables (e.g., global radiation, *etc.*) than PM_2.5_. *R* values for NO_2_ data were usually above 0.90 for training, validation and testing with error points which usually did not affect the time series of the data. Therefore, a reasonable prediction for exposure could be calculated over an annual average to make some estimates as to the health impacts in these working environments. The prediction of PM_2.5_ indoor air quality however, were considerably more varied with some *R* values for training, testing and validation of the networks below 0.53 ranging up to 0.97 (average *R* value = 0.819). Errors generally fell within the range of ±7 µg·m^−3^ although most are much less than this. Error points for PM_2.5_ had higher leverage causing the removal of peaks and troughs. This would ultimately affect the accuracy of the average exposure that could be calculated from such a modeled output. The *R* value decreases if training data is removed; when only test and validation data is calculated, the value decreased to 0.604 for validation and 0.779 for testing. For NO_2_ this value remained higher with *R* values of 0.893 for validation and 0.945 for testing.

The improvement of the prediction ability by the ANNs over use of best subsets regression can be seen at all sites. The *R* values significantly increased as hidden connections between the input data and the indoor concentrations were developed. The *R* = 0.952 (Table 2 and Figure S1) for testing of the newly trained NO_2_ network at M_c_2, while the best subset regressions for the same data set had found *R*^2^ values above 80% without using the hidden networks. Equally, an *R* value of 0.988 was attained for the ANN test data set for NO_2_ at M_c_3 indicating that a well-trained ANN had been developed using the meteorological variables and monitored outdoor concentrations of NO_2_ to predict indoor concentrations. This is compared to a best subset regression correlation of *R^2^* values of 79.9% for the same data set. For PM_2.5_ a low correlation at M_c_2 (*R*^2^ = 0.2) was found between indoor and outdoor concentrations when a best subsets regression was carried out prior to training of the ANN. This indicates that there was little direct interaction between indoor and outdoor concentrations and so other factors must have been influencing the indoor fluctuations. However, for the training, validation and testing the ANN produced an *R* value of 0.525. M_c_2 run 2 produced a much stronger trained network than run 1. M_c_2 run 2 outdoor monitoring was at ground level and the better-trained network may be due to the longer time that meteorological conditions have to influence the concentrations and therefore are more useful predictors. Challoner and Gill [3] previously found that ground level concentrations had a greater influence on indoor fluctuations than the roof level concentrations for this site. The prediction ability of *R* = 0.899 were strong compared to the original regression between indoor and outdoor values which had an *R*^2^ = 0.11 in M_c_2 run 2.

**Table 2:** Summary of Pearson *R* values for each run.

Site	Training	Validation	Test	All
NO_2_				
M_c_2 Run 1	0.999	0.988	0.967	0.991
M_c_2 Run 2	1.000	0.815	0.952	0.968
M_c_3 Run 1	0.996	0.994	0.988	0.994
M_c_3 Run 2	1.000	0.903	0.965	0.986
N_t_2 Run 1	0.977	0.804	0.956	0.968
N_t_2 Run 2	1.000	0.915	0.814	0.980
PM_2.5_				
M_c_2 Run 1	0.648	0.235	0.709	0.526
M_c_2 Run 2	0.985	0.781	0.776	0.900
M_c_3 Run 1	0.954	0.012	0.865	0.668
M_c_3 Run 2 (street)	0.984	0.969	0.925	0.951
PM_2.5_				
M_c_3 Run 2 (roof)	0.999	0.965	0.811	0.966
N_t_2 Run 1	0.984	0.631	0.666	0.844
N_t_2 Run 2	1.000	0.814	0.940	0.908

The significance of errors on the models depend upon when they occur-those which drag or push the time series away from its target trend-line are considerably more important than those which do not. In general, the errors found for the training of the ANNs, particularly for NO_2_ concentrations, did not all have high leverage on the data sets. Although many were large errors, the impacts on the data series trend-line were small, due to the positioning of the previous and proceeding data points. However, for the PM_2.5_ data, the errors did significantly influence the time series over an extended period of time, over-predicting for certain time periods and under-predicting for others (see for example M_c_3 run 1).

Finally, the simultaneous outdoor air quality monitoring at both roof level and ground level during run 2, PM_2.5_ at M_c_3 demonstrated how the ability of the ANN to predict the indoor monitored data was significantly improved.

### 3.2. Results from PALM Model

The PALM-GIS model was applied to the three inner city sites with the purpose of modelling the NO_2_ and PM2.5 outdoor concentrations for the “Run 2” periods (Table 3). The purpose of this modelling step is to provide a modeled input for the forward prediction of Indoor Data model presented in Section 4.

**Table 3:** Summary statistics for the PALM-GIS model for NO_2_.

Model Summary		
Building	*R*^2^	Std. Error
M_c_2	0.854	3.15
M_c_3	0.870	4.66
N_t_2	0.829	3.91

#### 3.2.1. NO_2_

The correlation between NO_2_ measured and modelled data (using PALM-GIS) is described in detail in the model summary statistics (Table 3) and analysis of variance (ANOVA) (Table 4) tables presented below. The coefficient of determination ranges between 83% and 87% means that the PALM-GIS model was able to predict with good accuracy the NO_2_ levels outside the selected buildings.

**Table 4:** Analysis of variance between measured and modelled data for NO_2_.

ANOVA						
Building	Model	Sum of Squares	Degrees of Freedom (DF)	Mean Square	F-Test	Significance Level
M_c_2	Regression	4357.6	1	4357.6	438.2	0
	Residual	745.9	75	9.95		
	Total	5203.4	76			
M_c_3	Regression	10,009.2	1	10,009.2	460.9	0
	Residual	1498.5	69	21.72		
	Total	11,507.6	70			
N_t_2	Regression	6980.9	1	6980.9	455.9	0
	Residual	1439.3	94	15.31		
	Total	8420.3	95			

#### 3.2.2. PM_2.5_

The correlation between PM_2.5_ measured and modelled data is described in detail in the model summary statistics (Table 5) and ANOVA (Table 6) tables presented below. The coefficient of determination ranges between 71% and 77%, revealing a lower correlation than for the NO_2_ cases. This might be due to the contribution from long-range sources of PM_2.5_, which is not explicitly accounted for in the PALM-GIS model.

**Table 5:** Summary statistics for the PALM-GIS model for PM_2.5_.

Model Summary		
Building	R^2^	Std. Error
M_c_2	0.711	2.17
M_c_3	0.760	2.06
N_t_2	0.770	1.85

**Table 6:** Analysis of variance between measured and modelled data for PM_2.5_.

ANOVA						
Building	Model	Sum of Squares	DF	Mean Square	F	Sig.
M_c_2	Regression	810.0	1	810.0	172.48	0
	Residual	328.7	70	4.696		
	Total	1138.7	71			
M_c_3	Regression	927.1	1	927.1	218.44	0
	Residual	292.9	69	4.244		
	Total	1220.0	70			
N_t_2	Regression	1071.6	1	1071.6	311.96	0
	Residual	319.5	93	3.435		
	Total	1391.0	94			

## 4. Forward Prediction of Indoor Data

### 4.1. Forward Prediction Using the Trained ANNs

The outdoor NO_2_ and PM_2.5_ air quality data as predicted by the PALM-GIS model at the three inner city sites for the “Run 2” periods of monitoring were entered as input data into the ANN models to forward predict the indoor air quality in these buildings. This has then been compared against the actual monitored indoor air quality.

#### 4.1.1. Results of Forward Prediction of NO_2_ Concentrations

The availability of monitoring data with two runs at the same monitoring locations left two opportunities to carry out a forward prediction for NO_2_, at M_c_3 (a recently constructed mechanically ventilated building) and N_t_2 (an older naturally ventilated building). Both sites showed different I/O ratios between the data for run 1 and 2 and a varying influence of meteorological parameters.

##### M_c_2 (Mechanically Ventilated Office)

**Figure 6:**
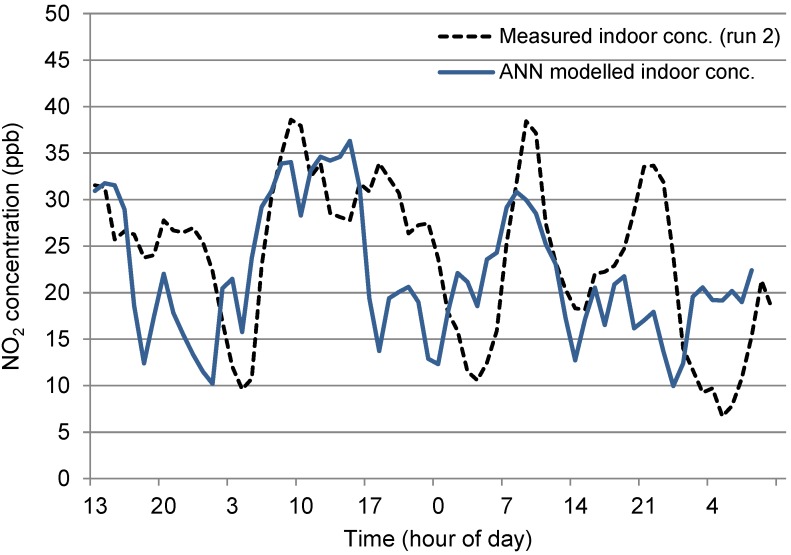
Measured *versus* modelled NO_2_ concentrations at M_c_2.

140 h of data were inputted into the model using indoor and outdoor concentrations from run 1 plus outdoor concentrations from run 2 (see Figures S1 and S2); these were supplemented by meteorological conditions for the two runs. Figure 6 shows the modelled concentrations of NO_2_ compared to the measured indoor concentrations. While the *R*^2^ correlation between measured and modelled indoor concentrations was only 0.14, a 2 Sample *t*-test of the indoor and predicted data gave reasonable result with a *t*-value = −1.51, *p*-value = 0.132, degrees of freedom (DF) = 129. The 95% confidence interval for the difference was (−4.68, 0.62). Hence, whilst the model does not predict the exact timings of the peaks and troughs in the monitored data, it does give a fairly accurate reflection of the average level of exposure throughout the day, which is of importance from a health perspective.

##### N_t_2 (Naturally Ventilated Office)

**Figure 7:**
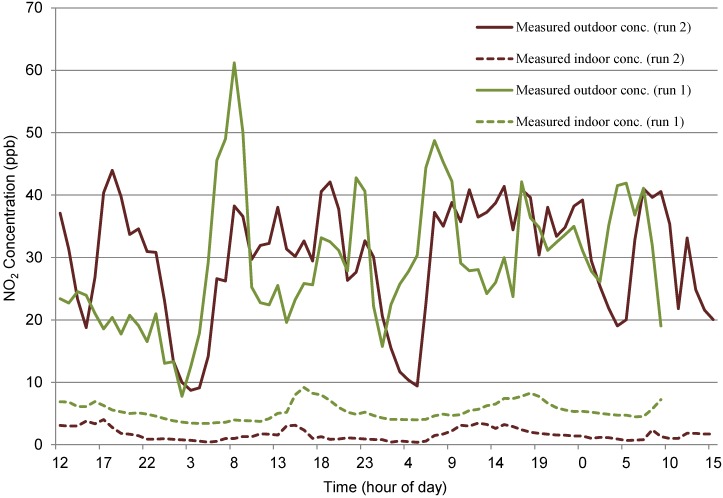
Measured indoor and outdoor NO_2_ concentrations at N_t_2 (run 1 and run 2).

**Figure 8:**
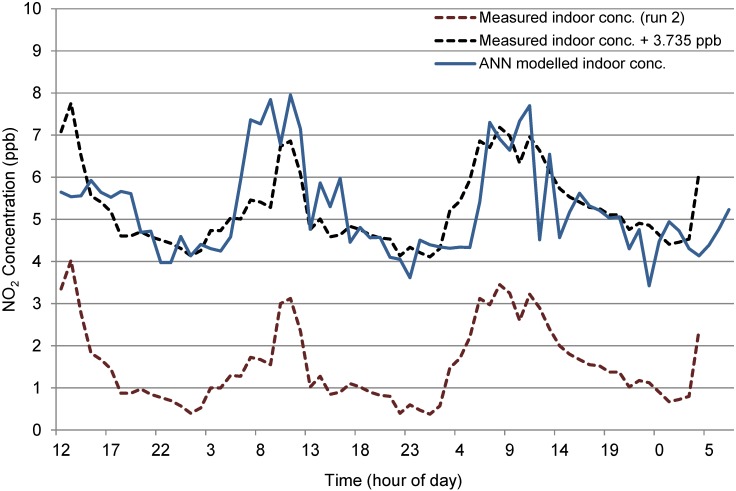
Measured *versus* modelled indoor NO_2_ concentrations at N_t_2 (run 2).

The outdoor data for site N_t_2 had comparable mean values for run 1 and run 2 (29.13 and 30.29 ppb respectively,) however, indoor concentrations revealed a greater difference in mean values (5.36 and 1.63 ppb respectively)—see Figure 7, with a varying start to the morning peaks giving the plots of both indoor concentrations a lagged effect. The difference in average indoor concentrations affected the ANN Model. As discussed previously, this reduction in concentrations indoors was due to a suspected increase in heterogeneous reaction rates indoors, which was not explicitly included as an additional input variable in the model. The modelled concentrations were therefore consistently higher than actual values for run 2, as shown in Figure 8, although the model did forecast relatively good predictions for the fluctuations. The difference in mean indoor concentrations (attributed the NO_2_ sink) over the run was 3.735 ppb which, if removed from each time step of the modelled values results in a much closer revised prediction, as shown Figure 8. Results from this adjusted model show a two Sample *t*-test give a 95% Confidence interval for difference: (−0.217, 0.494), *t*-value = 0.77, *p*-value = 0.443 and DF = 132.

#### 4.1.2. Results of Forward Prediction of PM_2.5_ Concentrations

As noted previously the relationship between pollutants for PM_2.5_ showed a much greater amount of variability compared to that for NO_2._ This led to weaker network predictions for PM_2.5_, and consequently poorer forward predictions using the trained network, as detailed below.

##### M_c_3 (Mechanically Ventilated Gallery Space)

**Figure 9:**
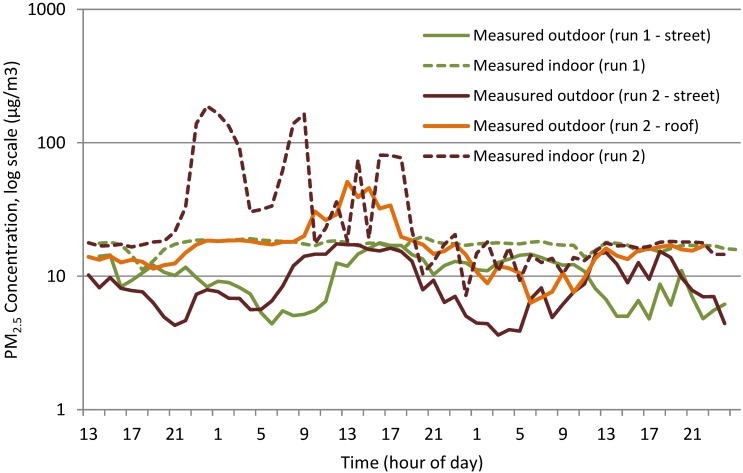
Measured indoor and outdoor PM_2.5_ concentrations at M_c_3 (run 1 and run 2).

**Figure 10:**
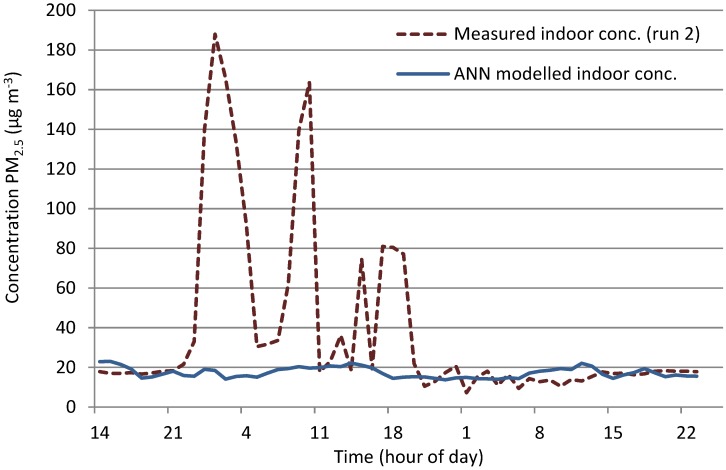
Measured *versus* modelled indoor PM_2.5_ concentrations at M_c_3 (run 2).

The relationship between indoor and outdoor for run 1 and run 2 differed significantly; with a considerable increase in indoor PM_2.5_ concentrations indoors during run 2. These peaks, as seen in Figure 9 on a log scale and Figure 10, were not picked up in ground level outdoor data, but were present at roof level. These indoor peaks during run 2, which were not present outdoors at ground level or apparently caused by a change in meteorological conditions, meant that it was not likely that the trained network would be able to anticipate their presence, as was the result shown in Figure 10.

While the model achieved the indoor value range for the beginning and second half of the data set, the peaks as shown in Figure 9 are not present and therefore, modelled data shows no indication of the peaks indoors (Figure 10). The use of outdoor roof level data, which showed reduced versions of peaks, may have improved the predictions but since only street level outdoor data was available for run 1, the network could not be trained using roof level data.

##### N_t_2 (Naturally Ventilated Office)

Figures S16 and S17 show the relationships between run 1 and 2 for indoor and outdoor concentrations for PM_2.5_ at Site N_t_2. Outdoor concentrations produced similar patterns with a clear diurnal pattern for both runs. Conversely, indoor concentrations did not show the same pattern (2 sample *t*-test results: 95% C estimate of difference (−4.850, −3.253), *t*-value = 10.1, *p*-value = 0.0, DF = 80) with run 1 having a considerably smoother pattern than run 2 and a higher mean. Again, as for the NO_2_ results at this site, this pattern appeared to be due to indoor variations rather than meteorological changes or a difference in outdoor concentrations, which the trained network did not incorporate.

**Figure 11:**
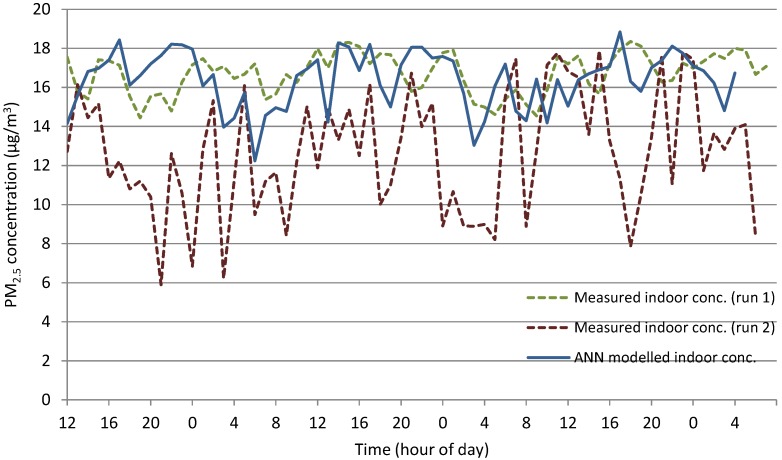
Measured PM_2.5_ concentrations *versus* modelled concentrations at N_t_2.

The forward prediction model was run using PM_2.5_ data from run 1 at Site N_t_2 as inputs, with the resultant output concentrations shown in Figure 11. A 2 Sample *t*-test found with 95% confidence that indoor run 1 and the modelled indoor run were not statistically significantly different (*t*-value = −1.37, *p*-value = 0.174, DF = 121). This indicates that the model may not be able to predict very short-term fluctuations, however it can predict a mean indoor value using the outdoor and met data that is statistically similar to the actual value. A further two Sample *t*-test was run to compare the modelled value and indoor run 2 concentrations. The results show that the two are significantly different statistically (*t*-value = 8.98, *p*-value = 0.000, DF = 92). This was expected as the two indoor runs vary in both magnitude and pattern.

### 4.2. Forward Prediction of a Generic Inner City Commercial Building

**Figure 12:**
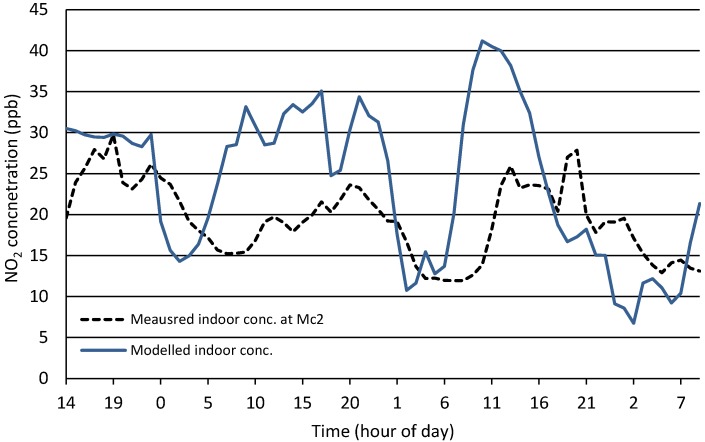
Modelled indoor NO_2_ concentrations at M_c_2 (using M_c_3) *vs.* measured indoor concentrations.

The forward prediction ability of the modelling approach was further assessed by using a trained ANN model from one site (M_c_3) to predict the indoor air quality at another site (M_c_2) of similar properties (*i.e*., both mechanically ventilated) using the inputs (outdoor pollutant concentrations and meteorological data) from the second site. Figure 12 shows that the results yielded a poor prediction with an estimate of the difference between the mean predicted indoor concentrations and actual concentrations of 4.58 ppb and 11.1%. Although these two buildings were similar; both built at the same time, located next to each other, and both with mechanical ventilation systems, other differences in building characteristics such as different uses and layouts were obviously not accounted for in the ANN model which had been trained to the characteristics of just one building.

## 5. Discussion

### 5.1. Forward Prediction Ability

The ANN modelling approach does show an ability to predict mean indoor NO_2_ exposure values from outdoor air quality data and ambient meteorological conditions for a given building, providing there is no other significant indoor production or degradation process occurring between the period where data is collected to train the network and the period for which the model is being used to make predictions. The ability of the model to predict PM_2.5_ however is much reduced. Improved predictions should be found if longer monitoring periods can be used to train the model, particularly if these include more variation in indoor and outdoor conditions. The ANN did show the ability to adapt to variations in the relationship between indoor and outdoor air quality. For example, at the end of run 1 at M_c_3 a change in the air pressure caused the relationship to change between indoor and outdoor which was similar to the relationship fluctuation seen in run 2. As the network was trained with run 1, the resultant forward prediction ability for indoor concentrations during run 2 was strong. This was not the case however, at N_t_2 where the run 1 data upon which the network was trained, did not seem to include the full dynamics between outdoor and indoor air quality that occurred during the second run. The relationship seen in run 2 showed a stronger sink between outdoor and indoor for NO_2_ with the results that the trained model was unable to correctly predict the level of indoor concentrations during run 2.

The ANN models also proved to be not so flexible when trying to transfer their indoor air quality predictions to other inner city buildings of apparently similar characteristics (on which they had not be explicitly trained) which indicates a significant limitation to the approach of this type of air quality modelling, based upon such limited monitoring data at least. It would obviously be infeasible to carry out detailed indoor and outdoor air quality monitoring for all buildings of interest in order to develop appropriate models.

However, once trained, these networks can be used to predict future longer-term averages in indoor air quality concentrations in the monitored buildings using updated outdoor concentrations provided by the PALM-GIS model and weather data from ambient stations. In Ireland, the EPA does not monitor PM_2.5_ data at hourly intervals therefore forward prediction would only be applicable for use in conjunction with NO_2_, which is available in hourly resolution. However, the testing of PM_2.5_ for forward prediction using data from N_t_2 showed a poor result indicating that even if hourly data was available it is unlikely to predict indoor pollutant exposure sufficiently.

It is interesting to note that interviews with building occupants showed an enthusiasm to learn about their air pollutant exposure levels. Hence, a future application of this work could be online tool or phone application to give building occupants indicative indoor concentrations. This would require a robust data base of generalized building types trained with a forward prediction model which could be linked with an online tool. Linking with real time traffic information and metrological data has the potential to give real time data feeds. Such a generalised model could realistically be fully developed for NO_2_ but maybe not for PM_2.5_ due to the model’s apparent poor ability for forward prediction. However, the WHO has previously stated that NO_2_ is strongly correlated with other toxic traffic related pollutants, such as benzene and toluene. Therefore, NO_2_ could be used as a surrogate to indicate concentrations of various other pollutants.

### 5.2. Implications to Public Health

The quality of air is rarely, if ever, considered when choosing a place of work, yet poor air quality will significantly affect the quality of health enjoyed by the employees. The average human inhales 20,000 litres of air daily or 14 litres per minute increasing to 50 litres per minute under intense physical exercise [40]. Over the past two decades strong evidence has been gathered showing links between fine particulate matter and respiratory/cardiovascular illnesses [14,41,42,43,44,45]. These illnesses include asthma, acute bronchitis, lung cancer, damage to nasal passages and respiratory tract inflammation. Previous research [46], noted that even a 2 µg m^−3^ difference in average exposure to PM_2.5_ over a life time in Dublin can reduce the life expectancy of a person by 6 months. Recent indoor studies have also provided evidence of effects on respiratory symptoms among infants at NO_2_ concentrations below the annual mean 21 ppb limit [47]. Hence, the modelling approach as presented in this research can help to provide information as to realistic daily and longer-term exposures and thereby feed into debates surrounding new indoor air quality legislation.

The data presented here was part of a wider research project (see [3]) that was carried out on 10 inner city buildings (five mechanically ventilated, five naturally ventilated). This found that the indoor air quality in several of the buildings showed an exceedance of the WHO annual mean 21 ppb guideline value for NO_2_ [48] during averaged working hours, but no site exceeded the maximum 1 h NO_2_ concentration WHO guideline limit of 105 ppb. In general, naturally ventilated buildings showed lower NO_2_ concentrations indoors, than the mechanically ventilated buildings. The highest maximum 1 h values recorded indoors were at M_c_3 (run 2) of 38.6 ppb. An interesting feature from the indoor data at many sites was that the indoor NO_2_ concentrations only dropped to 10 to 12 ppb, particularly inside the mechanically ventilated buildings, even though outdoor concentrations had dropped to much lower levels. Outdoor roadside NO_2_ concentrations at the 10 monitored sites had an average concentration at 22.4 ppb and a max 1 h concentration of 79.6 ppb in heavily trafficked areas of Dublin city centre. For comparison, the European average for trafficked sites in 2008 was found to be 43.2 ppb, almost double the average roadside concentration found in Dublin [49]. Equally, a study in Osaka, Japan found average winter concentrations of NO_2_ of 53 ppb and summer time concentrations of 49 ppb for urban monitoring.

For PM_2.5_ there is no outdoor 1 h or daily limit under EU legislation currently, but an annual mean limit of 25 μg·m^−3^ has been set out by the CAFE directive [50]. The mean indoor PM_2.5_ concentration in the naturally ventilated buildings during working hours was 24.2 ± 8.5 μg·m^−3^, compared to 18.9 ± 6.2 µg·m^−3^ during non-working hours. Equally, in the mechanically ventilated buildings the mean indoor PM_2.5_ concentration during working hours was 23.7 ± 9.2 µg·m^−3^, compared to 20.9 ± 12.0 µg·m^−3^ outside working hours. Five sites were found to exceed the annual mean 25 µg·m^−3^ PM_2.5_ objective value during working hours.

This combined modelling approach of developing trained ANNs for specific inner city buildings, which are then fed by realistic outdoor concentrations at that street in the city from the PALM-GIS model could be used to provide a reasonable estimate of long-term indoor air quality in such workplaces. Such data can then be used to make assessments of public health given the amount of time an average person spends indoors at their workplace; it has been estimated, for example, that up to 75% of daily NO_2_ exposure occurs during working hours [51]. This modelling approach could also be used to assess how different building types, sites and other operational characteristics may act to either enhance or mute the ingress of outdoor pollutants into such working environments, which will be of interest to urban planners, architects and engineers in the future.

## 6. Conclusions

The ANN predictions showed stronger predictive abilities for indoor NO_2_ concentration fluctuations when compared to PM_2.5_ using outdoor concentrations, with meteorological variables. This was attributed to the more uniform NO_2_ diurnal patterns which are influenced by meteorological variables such as global radiation to a much greater extent than PM_2.5_.

Use of the forward predictions for NO_2_ showed an ability of the ANN model to accurately predict mean exposure values as long as similar meteorological conditions occurred to the data set that the model was trained upon. If longer monitoring periods, which covered a variety of meteorological conditions and indoor/outdoor relationships, were used in order to initially train the network, errors may be reduced.

Unfortunately, it was found that the ANN could not use a network trained using data from one site to predict indoor concentrations at another site. This was due to the differences in various buildings relationships between indoor and outdoor concentrations. Hence, its use as a predictive model may be somewhat limited and only applicable to sites which have gathered detailed indoor and outdoor air quality data previously.

Finally, the study has shown that the greatest influence on the quality of indoor air for the majority of buildings was the quality of outdoor air. Hence, once outdoor air is at a standard which protects human health, the implication is that indoor air will more than likely be close to this level. The monitoring undertaken for this paper was short term in nature but indicates that the air quality in Dublin is within EU limit values.

## Supplementary Files

Supplementary File 1
