# Supplementary material for: Prediction of Indoor Air Exposure from Outdoor Air Quality Using an Artificial Neural Network Model for Inner City Commercial Buildings
Source: Int J Environ Res Public Health. 2015 Dec 1;12(12):15233–53. doi: 10.3390/ijerph121214975 (PMC4690911; doi:10.3390/ijerph121214975)
Supplement: Supplementary File 1 [file ijerph-12-14975-s001.pdf]

Prediction of indoor air exposure from outdoor air quality using an artificial neural network model for inner city commercial buildings

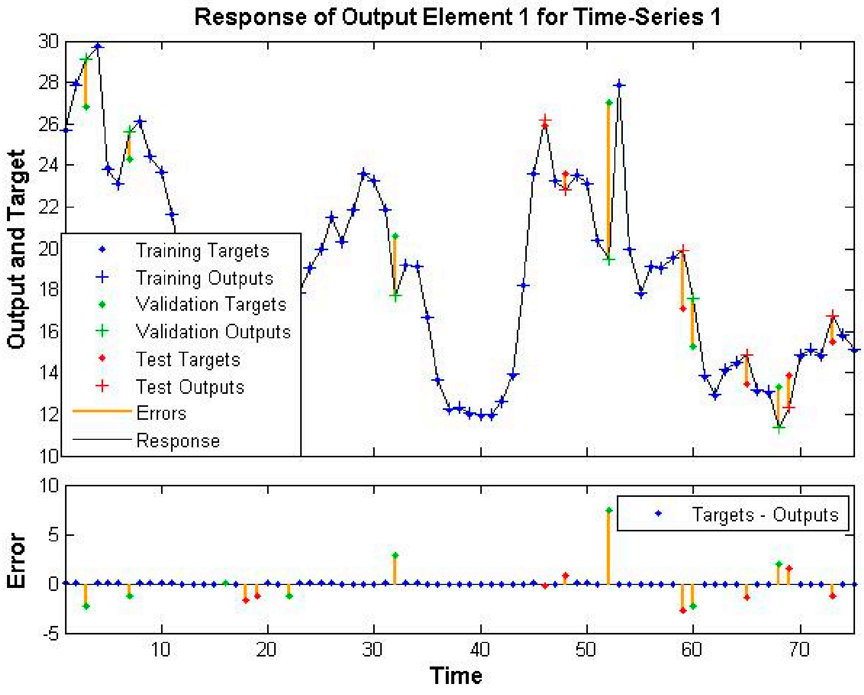

Figure S1. Time series of neural network training M_c2 run 2 NO_2.

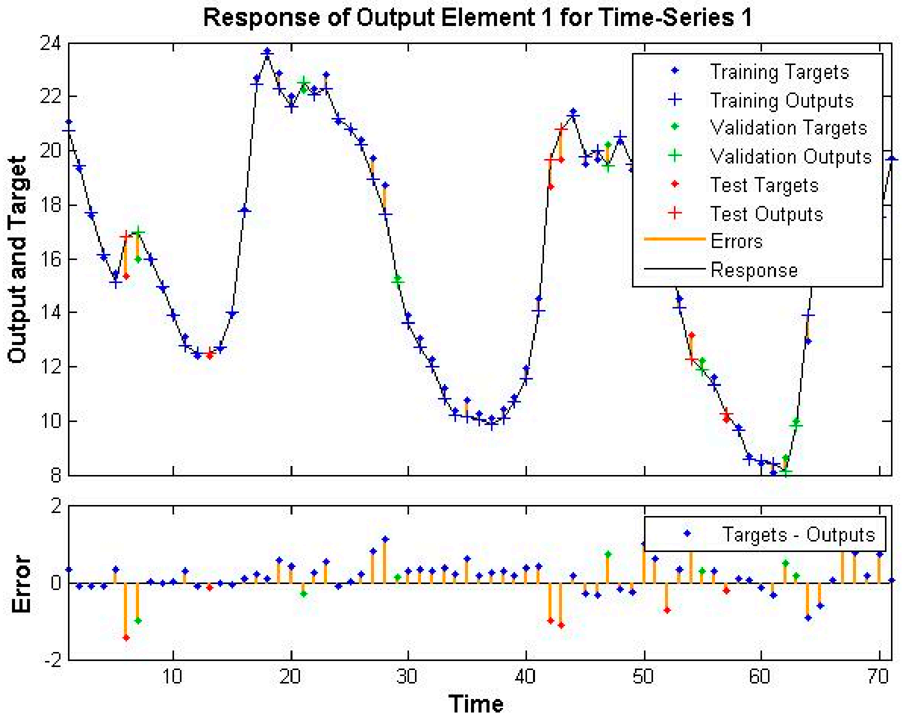

Figure S2. Time series of neural network training M_c3 run 1 NO_2.

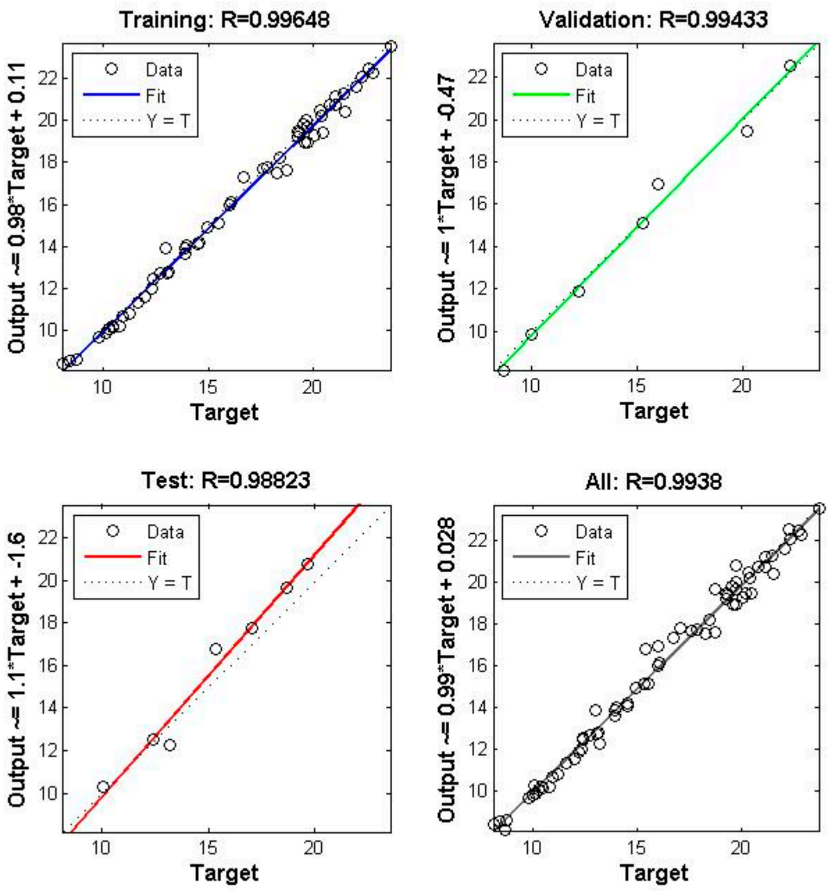

Figure S3. M_c3 run 1 NO_2 regression of trained output data set.

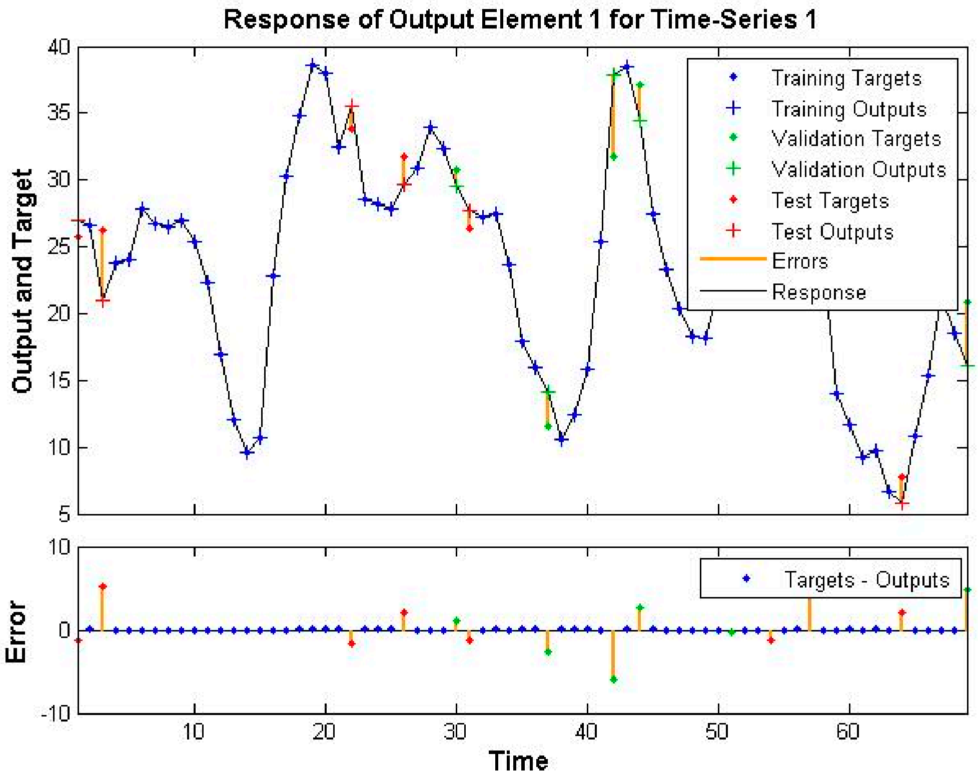

Figure S4. Time series of neural network training M_c3 run 2 NO_2.

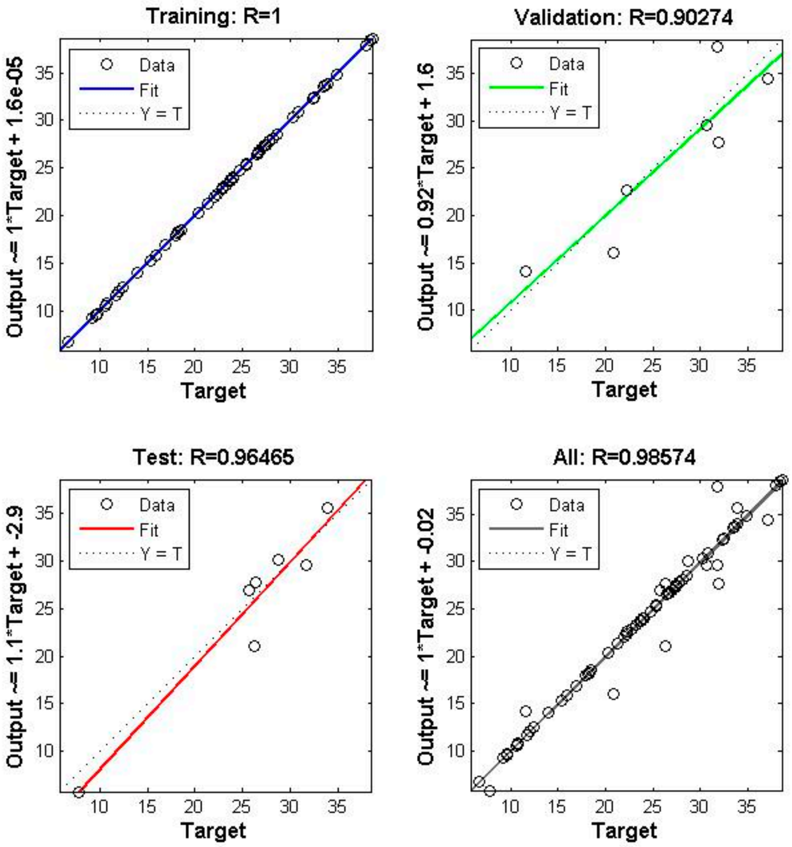

Figure S5. M_c3 run 2 NO_2 regression of trained output data set.

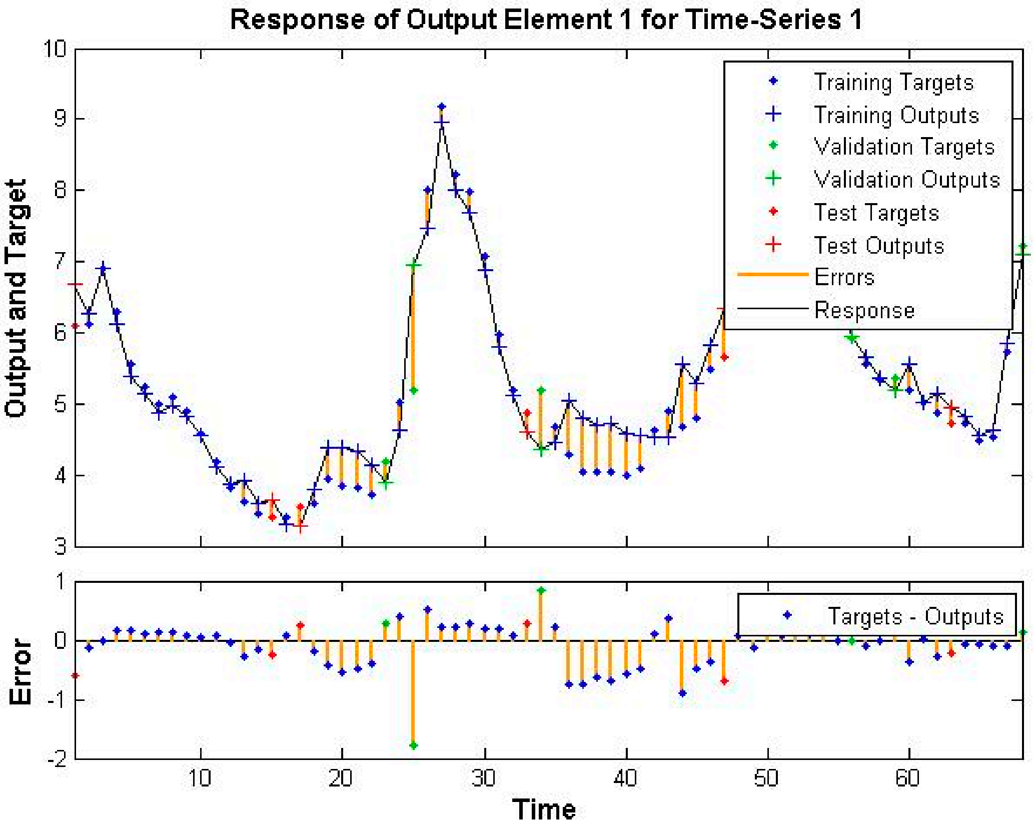

Figure S6. Time series of neural network training N_t2 run 1 NO_2.

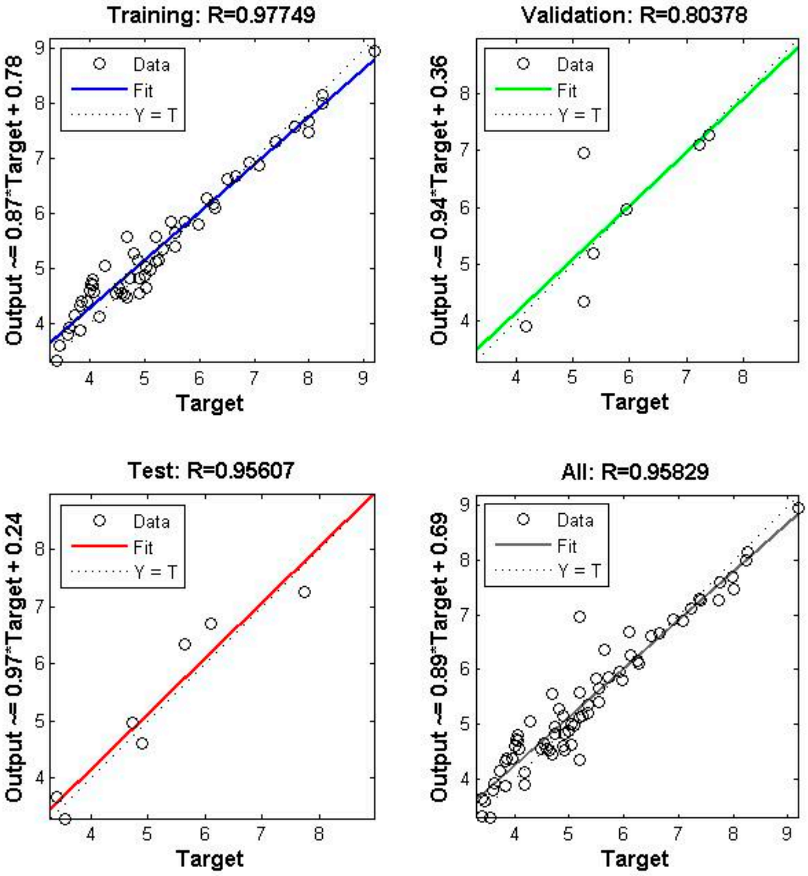

Figure S7. N_t2 run 1 NO_2 regression of trained output data set.

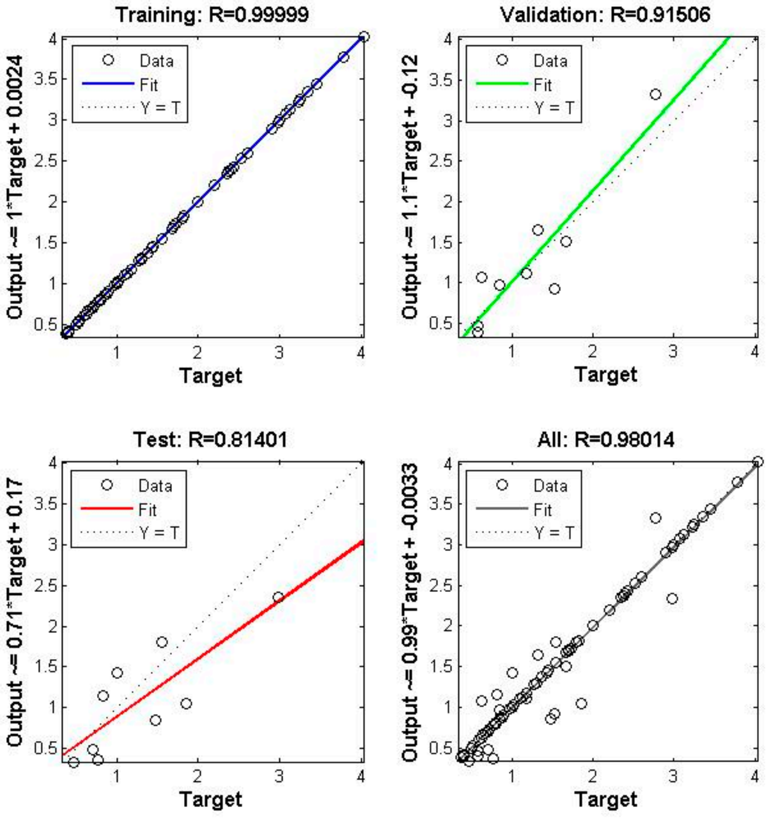

Figure S8. N_t2 run 2 NO_2 regression of trained output data set.

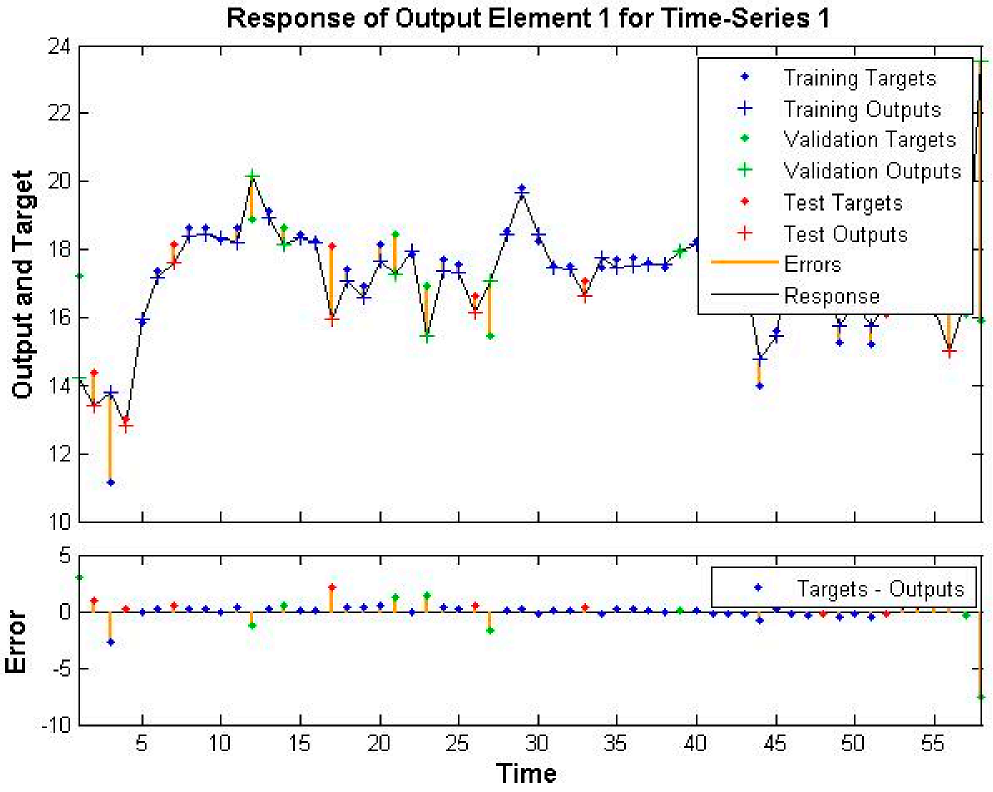

Figure S9. Time series of neural network training M_c3 run 1 $PM_{2.5}$.

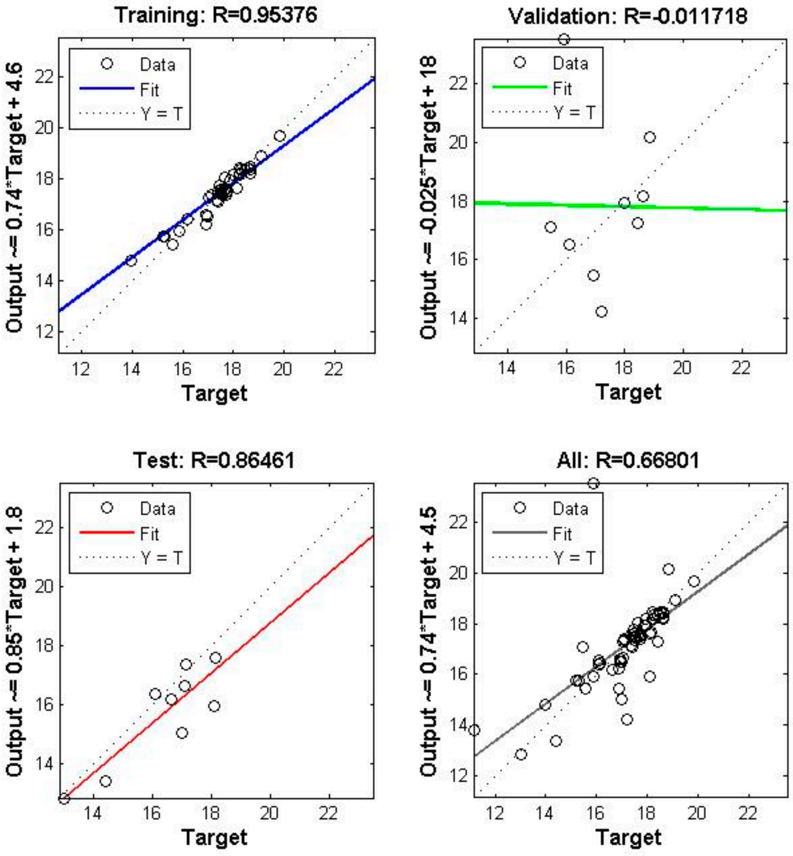

Figure S10. M_c3 run 1 $PM_{2.5}$ regression of trained output data set.

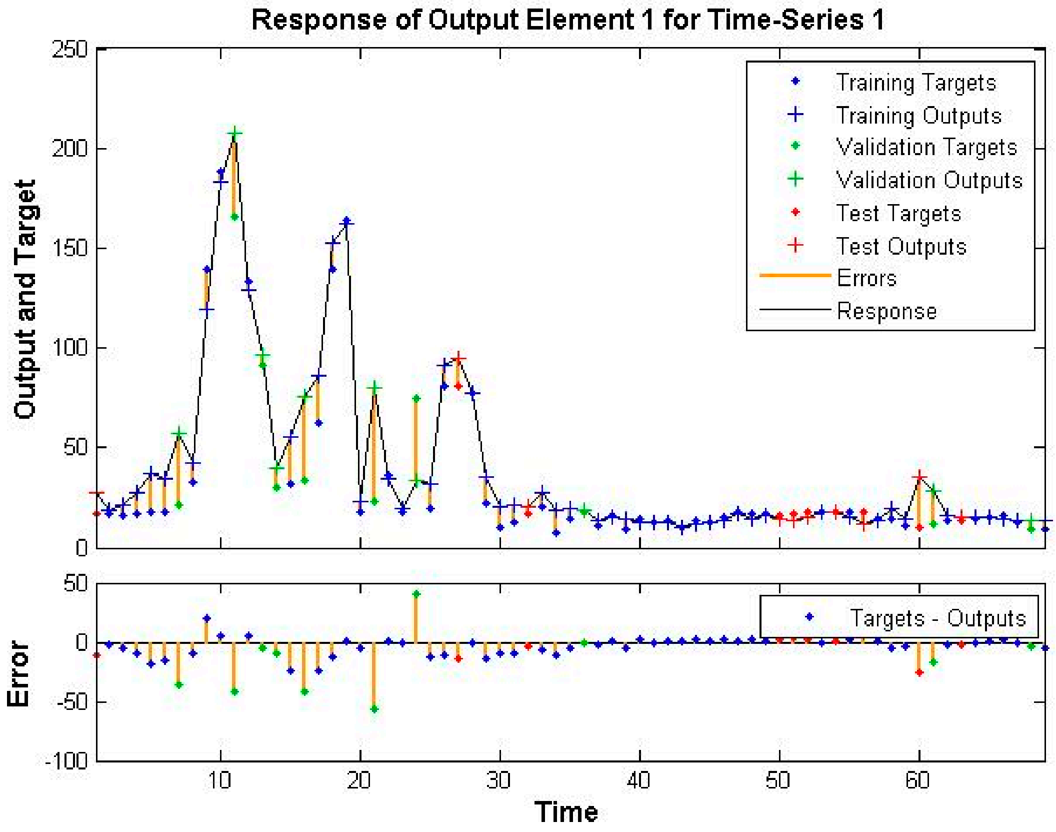

Figure S11. Time series of neural network training M_c3 run 2 (Street) $PM_{2.5}$.

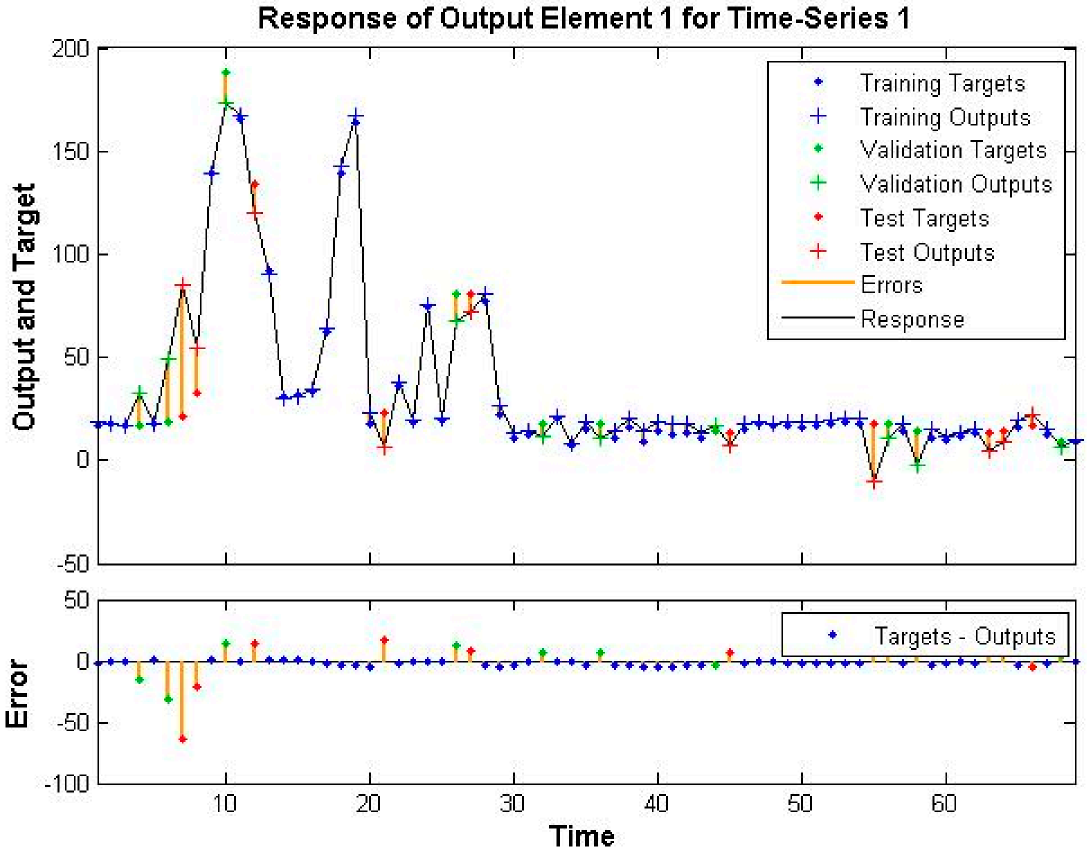

Figure S12. Time series of neural network training M_c3 run 2 (roof) $PM_{2.5}$.

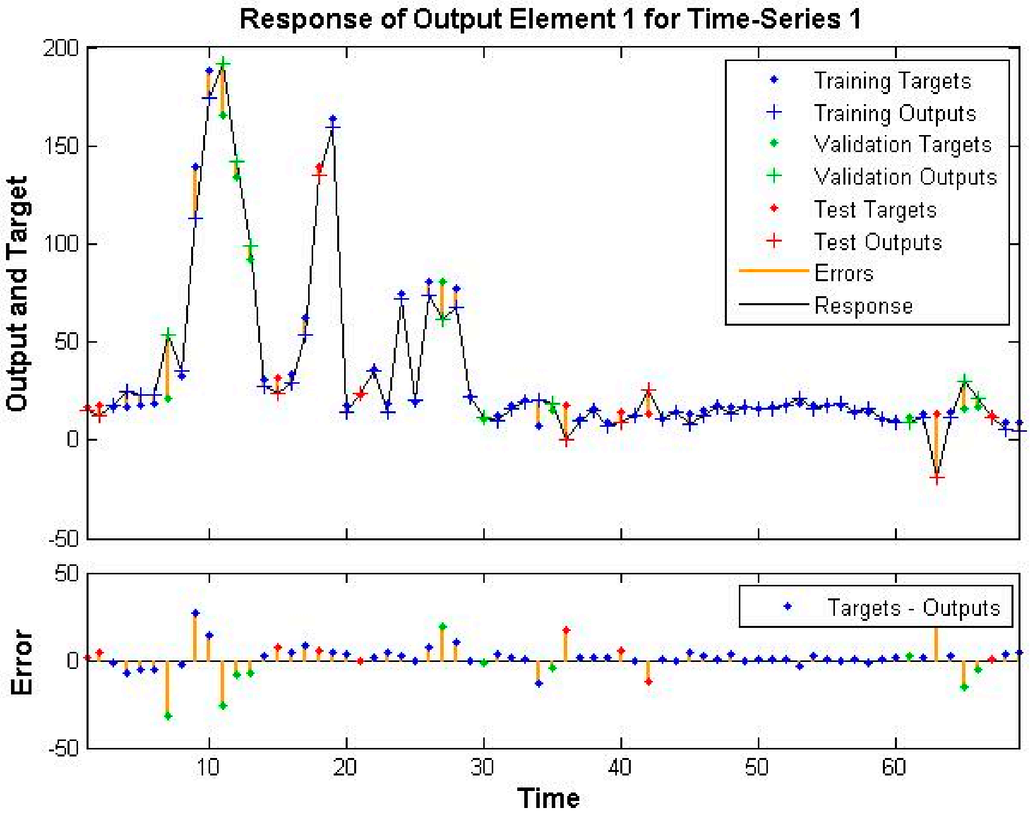

Figure S13. Time series of neural network training M_c3 run 2 $PM_{2.5}$.

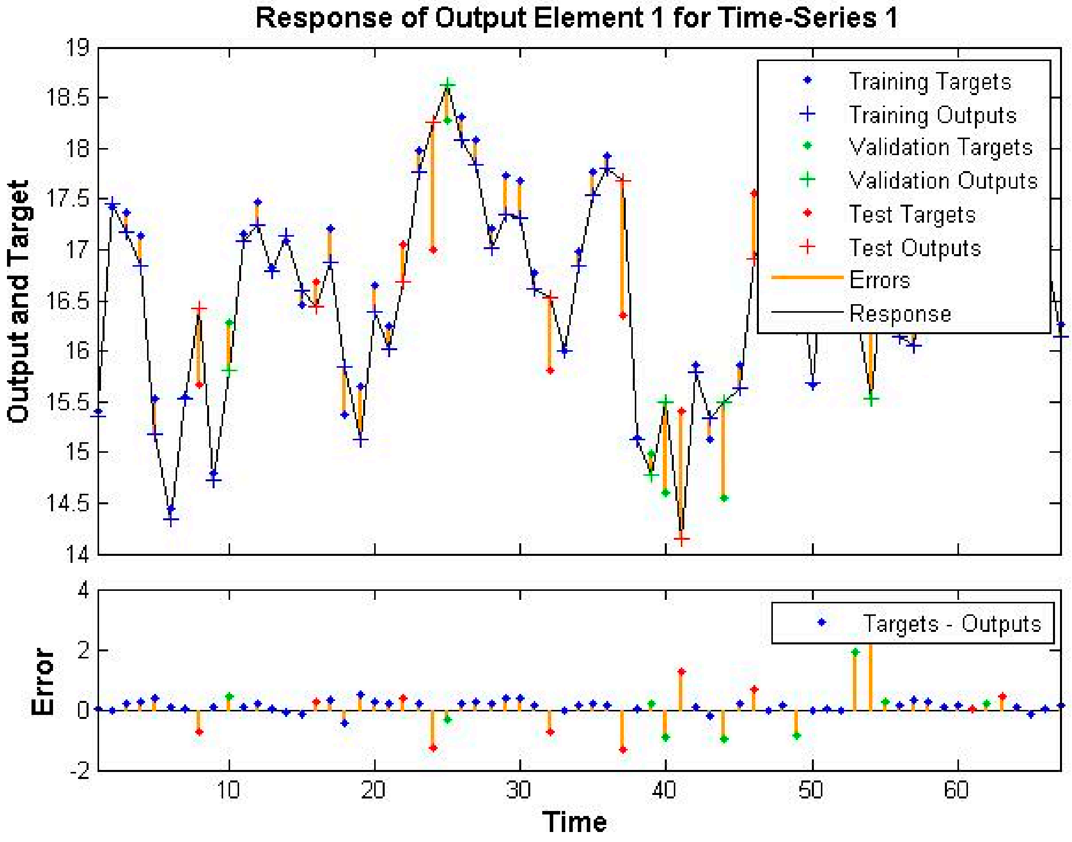

Figure S14. Time series of neural network training N_t2 run 1 $PM_{2.5}$.

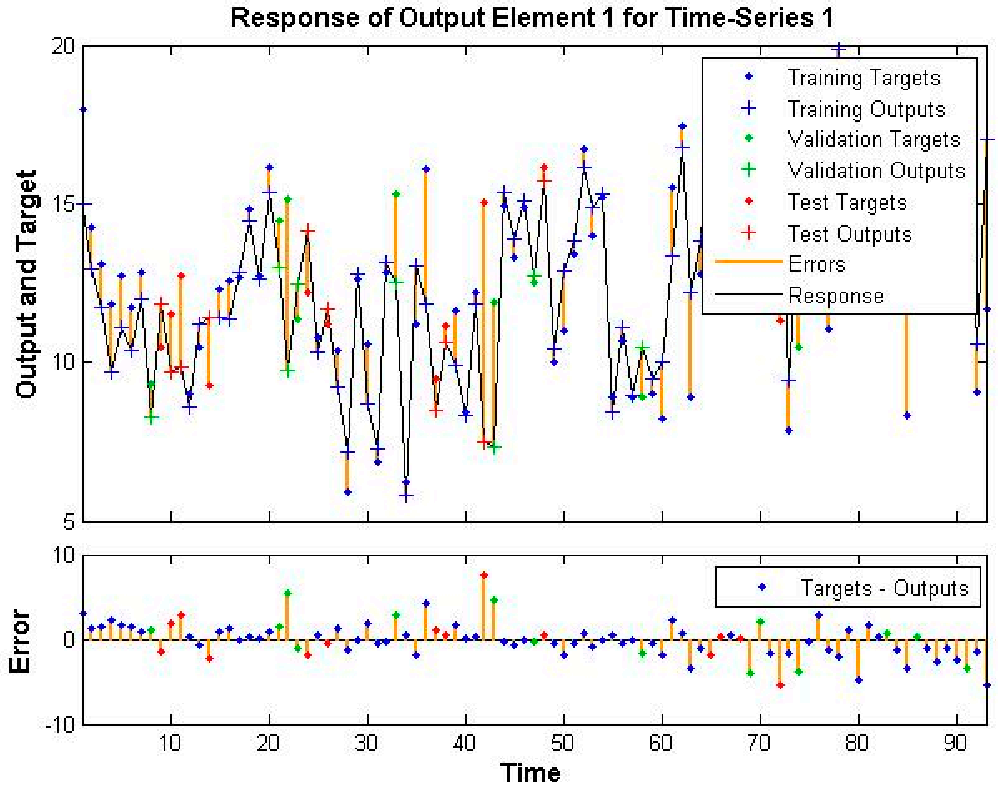

Figure S15. Time series of neural network training N_t2 run 2 $PM_{2.5}$.

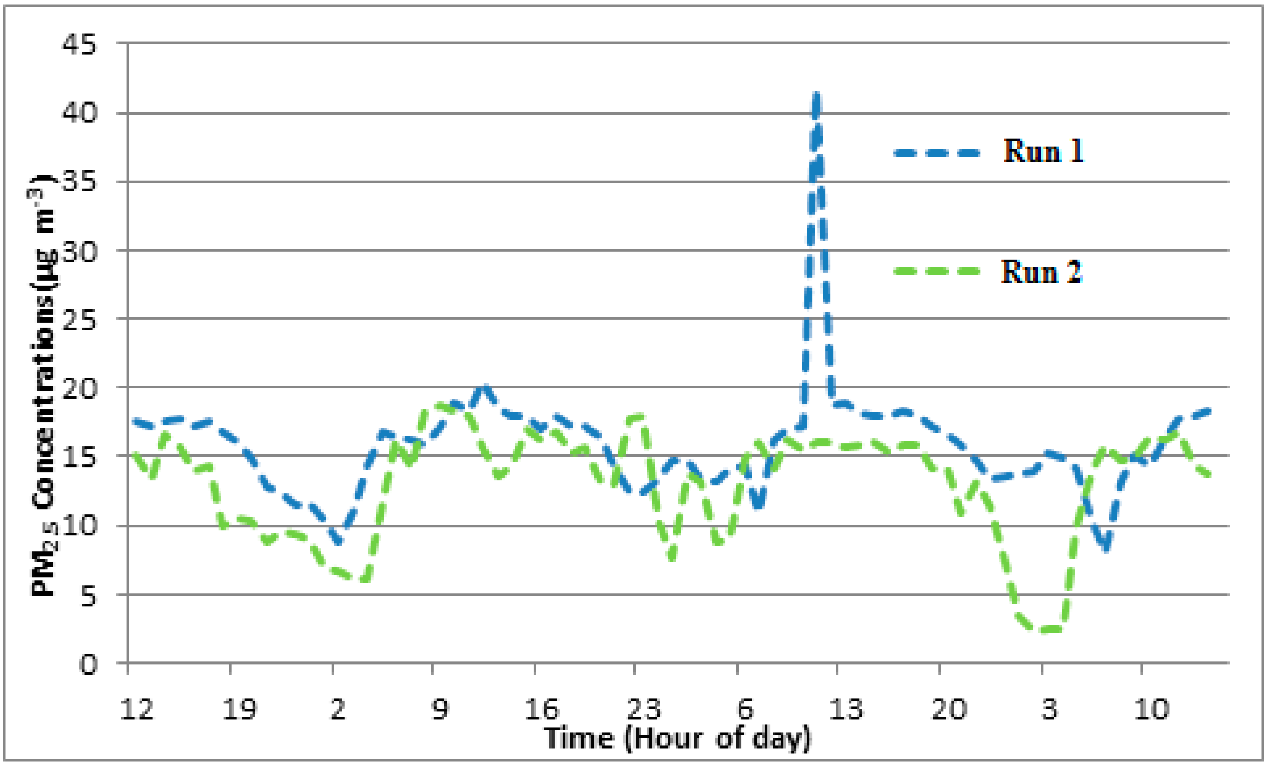

Figure S16. $PM_{2.5}$ outdoor concentrations N_t2 run 1 and run 2.

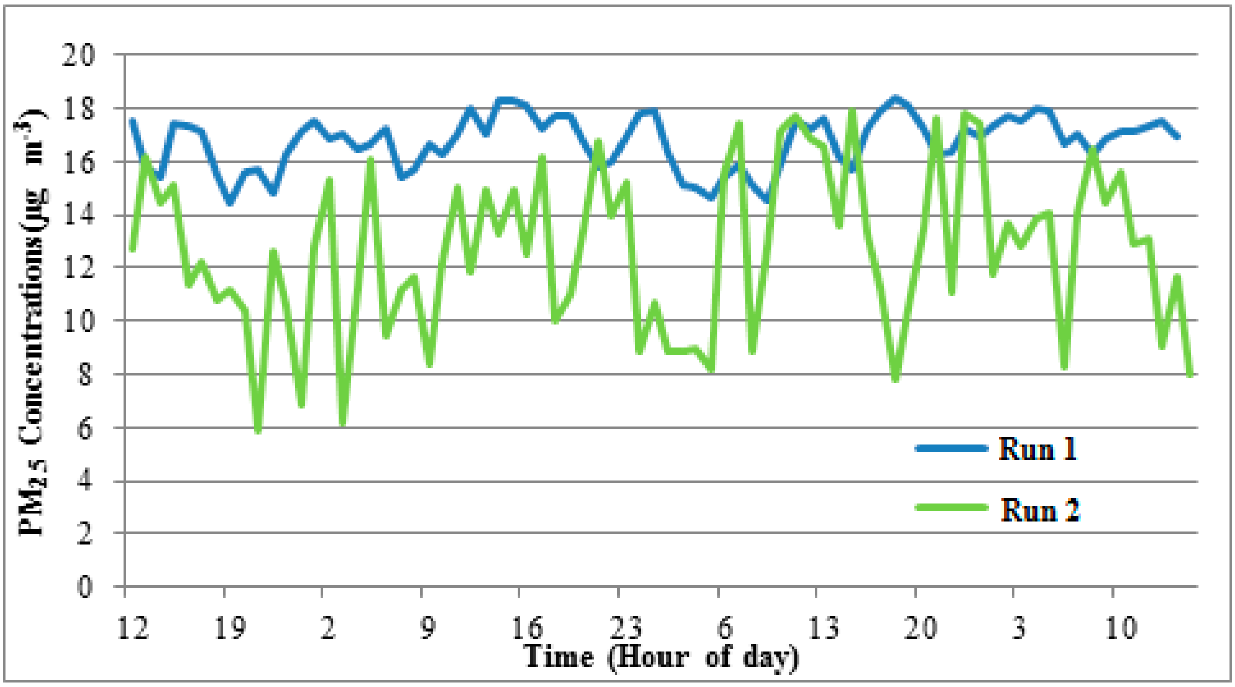

Figure S17. $PM_{2.5}$ indoor concentrations N_t2 run 1 and run 2.

© 2015 by the authors; licensee MDPI, Basel, Switzerland. This article is an open access article distributed under the terms and conditions of the Creative Commons Attribution license (http://creativecommons.org/licenses/by/4.0/).
